# Electrochemistry of Thin Films and Nanostructured Materials

**DOI:** 10.3390/molecules28104040

**Published:** 2023-05-11

**Authors:** Grzegorz Dariusz Sulka

**Affiliations:** Department of Physical Chemistry and Electrochemistry, Faculty of Chemistry, Jagiellonian University, Gronostajowa 2, 30387 Krakow, Poland; sulka@chemia.uj.edu.pl

**Keywords:** thin films, nanostructured materials, electrochemistry, cathodic and anodic processes, electrochemical characterization

## Abstract

In the last few decades, the development and use of thin films and nanostructured materials to enhance physical and chemical properties of materials has been common practice in the field of materials science and engineering. The progress which has recently been made in tailoring the unique properties of thin films and nanostructured materials, such as a high surface area to volume ratio, surface charge, structure, anisotropic nature, and tunable functionalities, allow expanding the range of their possible applications from mechanical, structural, and protective coatings to electronics, energy storage systems, sensing, optoelectronics, catalysis, and biomedicine. Recent advances have also focused on the importance of electrochemistry in the fabrication and characterization of functional thin films and nanostructured materials, as well as various systems and devices based on these materials. Both cathodic and anodic processes are being extensively developed in order to elaborate new procedures and possibilities for the synthesis and characterization of thin films and nanostructured materials.

## 1. Introduction

Typically, thin films are two-dimensional materials with a thickness ranging from a few nanometers to several micrometers. Despite the fact that humanity has been able to assess the thickness of these films only relatively recently, the history of making thin films is long. The first thin films made of Au were prepared in Egypt over 5000 years ago during the middle bronze age for decorative and optical applications [1,2]. For instance, Au films less than 300 nm thick covering copper and bronze statues and jewelry have been found in ancient tombs in the Pyramid of Djoser (Netjerykhet), built by the second King of the Third Dynasty, Old Kingdom (from 2667 to 2648 BC) in Saqqara [3]. Since then, humanity has learned not only how to obtain thin films of metals, metal oxides, various nitrides, carbides, semiconductors, and polymers [4,5,6,7,8], but also to study and control their chemical, optical, electrical, and mechanical properties, which differ considerably from the corresponding bulk materials [9,10,11,12,13]. The film properties can be precisely controlled by changing the thickness, which is a great benefit for thin film technologies and applications. Moreover, thanks to the development of technology, we are able to control the chemical composition, crystalline structure, electrical conductivity, thickness, and surface geometry of thin films [14,15,16,17]. It is known that the atomic structure, composition, morphology, surface roughness, microstructure (crystallinity and phases), defects or interfaces, and consequently, properties of thin films are closely related with the growth mechanism, parameters used for the synthesis (i.e., temperature, time, vapor pressure, gas mixture, precursor type, etc.), and deposition techniques [18,19]. Therefore, the resulting thin films can be of high quality, compact or porous, rigid or flexible, and amorphous, polycrystalline, or epitaxial in nature. Another important factor related to the synthesis of thin films is the type of substrate used for deposition and its preparation. The most commonly used substrates are metals, semiconductors (e.g., Si, conductive glasses ITO, FTO), aluminum oxide, and others. The substrate preparation procedure typically involves polishing/roughening, cleaning and chemical modification of the surface in order to provide a substrate for the deposition of thin films with the desired properties. Since thin films are often exposed to environmental factors such as chemical (pH, dissolved oxygen), radiative, and thermal stresses, the properties of thin films are usually designed and tailored for specific applications and are closely related to the method of thin film synthesis.

Currently, thin films can be obtained cheaply, reliably, and very efficiently using both physical and chemical deposition methods operating at the optimal conditions. Physical vapor deposition (PVD) covers a group of surface coating techniques, the most important of which include vacuum evaporation, [20,21,22], molecular beam epitaxy (MBE) [23], sputtering [22,24], and pulse laser deposition (PLD) [22,25]. Other physical techniques for the synthesis of thin films include spin coating [26] and spray coating methods [27]. Chemical vapor deposition (CVD) techniques are frequently used to obtain thin films from a variety of materials. Various CVDs also use organometallic precursors (MOCVD) [22,28], atomic layer deposition (ALD) [29], and electrodeposition [30,31,32,33,34].

The applications of thin films are very extensive and cover metallic conductive layers in silicon integrated circuits and integrated electronics [24,35], thin layers covering glasses in smart windows that transmit visible light, and reflect UV and infrared light [21,36], thin foils for magnetic recording, data storage, and logic circuits [22,37,38], transparent semiconductor layers in solar cells and photovoltaics [15,39], thin films in Li-ion batteries and supercapacitors [40,41], thin catalytic and electrocatalytic films for water electrolysis, wastewater treatment, and detecting chemical compounds [42,43], thin films protecting against corrosion, friction, and wear on parts of car and aircraft engines (pistons, cylinders, turbine blades, etc.) [44,45], and many others [46,47].

The use of nanostructured materials, though without consciously naming them, has been known in history for a long time. The Romans used Au nanoparticles to decorate glasses and cups (e.g., the Lycurgus cup with Ag and Au nanoparticles embedded in glass in the British Museum) as early as the fourth century A.D. [48]. Nanostructured materials have been of particular interest to scientists for several decades, since they were intentionally named. The first publication on the subject of nanostructured materials can be dated back to 1981, when Herbert Gleiter published a paper on the unique and exceptional properties of nanostructured materials [49]. Historically, nanostructured materials were defined as the finest microstructures with an average phase or grain size of the order of a nanometer (10^−9^ m), and in which 50% or more of the atoms are situated at grain boundaries [50,51,52]. Currently, the term is much more broadly defined and covers a wider range of materials, including materials that contain an internal or surface nanostructure. In general, nanostructured materials are defined as materials with at least one dimension in the nanoscale, i.e., in the range from 1 to 100 nm. Depending on the dimensionality (in which the materials are not nanoscaled) they are divided into nanoparticles (0D), filamentary structures (nanotubes, nanowires, nanorods, nanofibers) (1D), layered or lamellar structures (nanosheets, thin films) (2D), and bulk nanostructured materials (3D) [53,54,55]. In recent decades, many interesting nanostructured materials with unique properties have been obtained, and it is worth mentioning a few particular examples: colloidal nanocrystals [56,57], buckminsterfullerene C_60_ [58], covalent organic frameworks (COFs) [59,60], carbon nanotubes (CNTs) [61,62], metal, semiconductor, and polymer nanowires and nanotubes [63,64,65], and nanoporous materials such as anodic aluminum oxide (AAO) [66,67,68], anodic titanium oxide [69,70,71], metals [72,73], zeolites [74], and others [75].

Nanostructured materials are usually synthesized with the thickness not exceeding a few micrometers. However, this is not the only feature that distinguishes them from bulk materials. More important are such features as a large surface to volume ratio, favorable transport properties, altered physical properties, and confinement effects resulting from the nanoscale dimensions comparable to the wavelengths of electrons and photons [76,77]. In many cases, the physical and chemical properties of materials in the nanoscale range vary greatly from properties of the same substance on a macro scale. It is widely known that nanostructured materials show very high strength, reduced sintering temperatures, increased diffusivity, improved charge storage and transfer on the material surface, enhanced adsorption and catalytic activity, and attractive physical properties (e.g., light absorption and scattering properties, optical sensitivity, wettability) [76,78]. The improved physicochemical properties of these materials are the driving force behind the huge progress in the field of nanostructured materials. Not only materials science, but also other scientific disciplines (chemistry, physics, biology, medicine, etc.) are actively involved in research on nanostructured materials due to the different synthetic methods used and the wide variety of physical techniques adopted for their characterization.

In general, there are two basic strategies to synthesize nanostructured materials, and they do not differ significantly from those used for decades in materials science. The first group of methods includes the so-called top-down approaches, which involves reducing the size of bulk materials to nanoscale through the use of mechanical processes and lithography. Among them can be distinguished mechanical alloying [79], high-energy ball milling [80], equal-channel angle pressing [81], high-pressure torsion [82,83], accumulative roll bonding [84], chemical etching [85], laser ablation [86], and various lithography techniques [87,88,89]. Top-down methods of synthesis of nanostructured materials allow us to produce ideal periodic structures without any defects and in a repeatable manner, but often require advanced and expensive equipment, e.g., ion beam, E-beam, X-ray, and interference lithographic techniques.

For this reason, a second so-called bottom-up approach is widely used for the synthesis of nanostructured materials. These methods are attracting a growing interest because they ensure low costs and scalability of materials processing, and are suitable for the production of various nanostructured materials on an industrial scale. In the bottom-up approach, individual atoms or molecules are combined into larger objects using gas phase synthesis and wet chemical methods, which include inert gas condensation [90], physical/chemical vapor condensation [91], electrodeposition [92], electroless deposition or galvanic displacement (cementation) processes [93,94], co-precipitation [95], sol–gel [96,97], solution combustion synthesis [98], hydrothermal [97,99], spray pyrolysis [100], thermal evaporation [101], and plasma synthesis techniques [102].

A separate group of methods used for the synthesis of various nanostructured materials is a template-assisted synthesis, which was recently found to be an elegant, inexpensive, and relatively simple approach [103,104,105,106]. These methods use a variety of templates, including stepped templates (silicon, highly oriented pyrolytic graphite-HOPG), soft templates (surfactants, reverse micelles, colloids, polymers), nanoporous templates (mica, silicon, track-etched polymers, anodic aluminum oxide-AAO, block copolymers), and biotemplates (crystalline surface layers of bacterial cells, ferritin and ferritin-like protein cages, various biomolecules with a linear morphology such as DNA, viruses, microtubules, and lipid nanotubes). Templated methods, especially exploiting nanoporous templates, allow us to produce periodically ordered structures, including highly ordered and closely packed arrays of nanopores, nanodots, nanotubes, and nanowires. The main structural parameters of nanomaterials (e.g., diameter and aspect ratio being the length to diameter ratio) are controlled by the shape and dimensions of the template, as well as the operating conditions used for the deposition. A great variety of previously described physical and chemical deposition techniques are used to fill templates.

Extensive research on nanostructured materials and their characterization have shown their uniqueness and advantages for applications in various fields of science and engineering including chemistry, physics, biology, biotechnology, and medicine. Typical applications are related to electrochemical energy conversion and storage [100,107,108], including lithium-based batteries [109], supercapacitors [99,110], and fuel cells [111]. Energy-related applications of nanostructured materials cover also electrochromic energy storage systems [112], solar cells, and photovoltaics [113] as well as thermoelectric systems and devices [114]. Due to the expanded surface area and unique surface properties, nanostructured materials have also found applications in photo- and electrocatalysis [92,115,116]. In addition, various modern sensors and biosensors were produced on their basis [59,117]. There are many other new applications of nanostructured materials, and almost every day we can find in the literature some new applications and devices based on them.

This Special Issue of *Molecules* attempts to cover recent advances in electrochemical synthesis, characterization, and applications of diverse thin films and nanostructured materials. The papers of this collection can be organized in three main sections. There are two separate sections on the different types of synthesis process, namely cathodic and anodic processes. A separate section is devoted to electrochemical characterization of thin films and nanostructured materials.

## 2. Electrochemical Methods Used for the Synthesis and Characterization of Thin Films and Nanostructured Materials

Electrochemistry as a field of science provides, on the one hand, relatively simple methods for synthesis of various thin films and nanostructured materials, and on the other hand, analytical tools for electrochemical characterization, functionalization, and modification of novel materials, systems, and devices, as well as for the detection and determination of different analytes. Electrochemical methods of material synthesis are simple, cheap, do not require specialized and expensive equipment, and are easy to implement in any research laboratory or to scale up to an industrial scale. Both types of electrochemical processes, cathodic reduction and anodic oxidation, can be effectively used for synthesis of materials.

### 2.1. Cathodic Processes of Synthesis of Thin Films and Nanostructured Materials

Using cathodic deposition at different modes, such as constant potential, constant current density, pulsed electrodeposition or cyclic voltammetry (CV), various metals, alloys, metal oxides, semiconductors, conductive polymers, and even composites can be easily obtained, even on non-flat substrates. It is worth noting that a two-electrode configuration is used for the galvanostatic mode, while a three-electrode cell is used for the potentiostatic mode. Parameters used for cathodic deposition (e.g., electrolyte composition, deposition time, temperature, agitation of electrolyte, and applied current/potential) influence deposit properties (chemical composition, thickness, uniformity, and microstructure). The advantages of cathodic deposition of various materials include (i) the crystal nucleation and growth process can be controlled by the applied current density/potential, concentration of ions in the solution, and various additives; (ii) deposition of metal alloys and composite materials is possible, but the reduction potential of both components should be close enough to each other; and (iii) deposition is carried out directly on the conducting substrate.

The cathodic electrodeposition of metals from solutions in the form of thin films and coatings is well established. For many decades, these processes have been studied and improved to obtain decorative and protective coatings on various substrates. It is widely recognized that some additives present in the electrolyte used for electrodeposition may accelerate the process of metal layer formation. The influence of various substances, which can be present in the electrolyte, on the kinetics and mechanism of electrochemical copper deposition may be of great practical importance, because the obtained results can be used in the copper electrorefining process. The research presented in this Special Issue by Mroczka et al. [118,119] is of great interest in this context. The group of such functional additives includes SPS (bis-(sodium-sulfopropyl)-disulfide) and MPS (3-mercapto-1-propanesulfonate) in the presence of Cl^−^ ions [118], as well as SPS and polyethylene glycol (PEG) in the electrolyte with Cl^−^ addition [119]. The effect of these additives on their accumulation and incorporation in the copper thin layer has been studied using time-of-flight secondary-ion mass spectrometry (TOF-SIMS) combined with cyclic voltammetry measurements. It was found that accelerating the mechanisms of MPS and SPS in the presence of Cl^−^ is based on the ion pair interaction of sulfonate ends and Cu^2+^ that facilitates dehydration, followed by the reduction of Cu^2+^ to Cu^+^ and then transfer of Cu^+^ ions to the chloride adlayer [118]. The suppressing abilities of PEG in the electrodeposition Cu process is related to the formation of stable Cu-PEG adducts [119]. The inhibiting properties of PEG are reduced by the addition of SPS. It was concluded that the competition between SPS and PEG in capturing Cu^2+^ ions can be attributed to the higher affinity and flexibility of the sulfonate groups of thiolate molecules.

Unlike the electrodeposition of metals, a reductive electrochemical formation of thin, homogenous, and insoluble conductive polymer layers directly on the electrode surface is not a widely used method [120,121,122]. Typically, thin films of polymers derived from pyridyl and polypyridyl complexes containing styrene groups and complexes containing vinyl-substituted ligands are synthesized by electrochemical cathodic processes. Recently, Rendón-Enríquez et al. [123] proposed a reductive electrochemical method for the deposition of polymer films by cyclic voltammetry from 1,3,5-tris(2-chloropyrid-5-yl)benzene and 1,3,5-tris(2-bromopyrid-5-yl)benzene monomers dissolved in dimethylformamide (DMF). ITO substrates or platinum covered quartz crystals were used for deposition. The resulting polymers are considered to be potential electrochromic materials that can change color in response to an electrical stimulus. In addition, these polymer thin films are attractive for potential applications in photogalvanic or photovoltaic cells.

Cathodic processes are often used to obtain nanostructured materials, including, for example, metallic, composite, semiconductor, and other nanoparticles and nanowires [124,125,126,127,128,129,130]. This thematic area is covered by Ropero-Vega et al. [131] in their paper devoted to the deposition of Au nanoparticles (NPs) on screen-printed electrodes for potential application in electrochemical biosensors for the detection of *Escherichia coli* in aqueous matrices. The electrodeposition of AuNPs was carried out in 1.0 mM HAuCl_4_ and 0.5 M H_2_SO_4_ at a constant potential, and the influence of the applied potential (from 0.05 V to −0.25 V vs. Ag/AgCl) and deposition time (20–250 s) on the morphology of AuNPs deposited on the electrode surface was investigated. These studies optimized conditions for electrochemical cathodic deposition (−0.05 V vs. Ag/AgCl and 100 s) of AuNPs on the electrode surface.

### 2.2. Anodic Processes of Synthesis of Thin Films and Nanostructured Materials

Anodic oxidation is most often used as a simple and cost effective method for the formation of thin oxide layers on metals or conductive polymers on various substrates. The anodic oxidation of metals, known also as an anodization process, has been used over 100 years to produce protective and decorative layers on aluminum and its alloys [132]. A major breakthrough allowing the use of anodic aluminum oxide (AAO) in nanotechnology occurred after the initiation of the two-step anodizing procedure by Masuda and Satoh in 1996 [133], which resulted in the formation of highly ordered and densely packed nanoporous aluminum oxide layers. Since that time, huge progress in anodic oxidation of aluminum, characterization, and application of AAO has been made. The structural features of nanoporous AAO (pore diameter, interpore distance, layer thickness, pore density, and porosity) can be easily controlled by the anodizing conditions (electrolyte type and composition, applied potential, electrolyte temperature, and time). In recent years, anodization has also been adapted for the formation of nanoporous/nanotubular oxide layers on the surface of different metals such as Ti, W, Sn, Zn, Hf, Nb, Zr, and Mo [134]. In general, two review papers included in this Special Issue are devoted to AAO [135,136]. In the first one, Ono [135] reports how the electric field strength and ion incorporation, as factors other than voltage, affect the morphology and properties of AAO films. In this context, the pore initiation process, steady-state growth of porous structure, and sealing mechanism are reviewed. The effect of applied voltage/electric field strength on the structural features of AAO and incorporation of anions from electrolytes is discussed for typical electrolytes used for anodization of aluminum (i.e., sulfuric, oxalic, phosphoric, and chromic acids). Other aspects covered by this review include a radial branching of pores, defect formation mechanisms, coordination number of Al, and the formation of α-alumina membranes. The paper by Brudzisz et al. [136] is focused on the subject of anion incorporation in AAO, which occurs during anodization of aluminum. The incorporation of the electrolyte-derived anions (PO_4_^2−^, C_2_O_4_^2−^ and SO_4_^2−^) into the barrier layer and walls of AAO as well as its effect on optical properties of AAO (e.g., refractive index, photoluminescence, and galvanoluminescence spectra) are discussed in detail. In addition, the incorporation of other ions in anodic TiO_2_ is also reviewed briefly.

The anodization process is also used to produce a barrier coating on metals that, on the one hand, are biodegradable and can be used to produce bioimplants, and on the other hand, are subjected to rapid corrosion in body fluids. Magnesium is one such metal. Therefore, the processes of anodic oxidation of Mg in various electrolytes are studied, but very large potentials/currents are applied, so that discharges occur and the resulting plasma modifies the structure of the oxide layer. This process is called plasma electrolytic oxidation (PEO), and the resulting oxide layer forms a porous ceramic coating on the metal surface. Husak et al. [137] studied PEO of pure Mg in different silicate-based solutions with different concentrations of sodium silicate, sodium fluoride, calcium hydroxide, and sodium hydroxide. To form a ceramic coating on the Mg surface, a potential of 200–300 V was applied for 10 min. The corrosion properties of the new coatings were studied by long-term immersion in simulated body fluid (SBF). Biocompatibility and antibacterial properties of formed coatings were evaluated using U2OS cell culture and Gram-positive *Staphylococcus aureus* (strain B918). It was found that anodic oxidation of Mg in the proposed conditions results in stable and biocompatible oxide coatings with a high potential for commercial application as degradable implants.

Anodic oxidation can be also used for electropolymerization of conductive polymers with electrochromic properties, which are suitable materials for flexible photovoltaic modules, components of displays/screens and batteries, electrochromic windows, or photocatalysts [138]. The collection of articles in this Special Issue includes a paper on oxidative electropolymerization of different conducting polymers on FTO glass substrates by cyclic voltammetry [123]. The publication presents not only the electrosynthesis of such conductive polymers as poly(4,7-dithienyl-2,1,3-benzothiadiazole) (PDTBT), poly(3,4-ethylenedioxythiophene) (PEDOT), polythiophene (PT), polyaniline (PANI), and polypyrrole (PPy), but also a potential window of polymers useful for electrochromic applications.

### 2.3. Electrochemical Characterization of Thin Films and Nanostructured Materials

Electrochemical techniques have been used for many years to study various materials and electrodes, as they provide practical information in various areas of applications. Very often, potential sweep methods, especially linear sweep or cyclic voltammetries (LSV and CV), are used as simple, flexible routine techniques to study the kinetics of diverse electrode processes occurring on the surface of the tested materials/electrodes and also to characterize the redox properties of molecules. Other frequently used electrochemical techniques in electrochemistry and electroanalysis include step techniques (chronoamperometry, chronopotentiometry) and pulse voltametric techniques such as normal pulse voltammetry (NPV), differential pulse voltammetry (DPV), and square wave voltammetry (SWV) [139]. To investigate various electrochemical systems and processes, a non-invasive technique known as electrochemical impedance spectroscopy (EIS) can be also used. It allows us to study bulk and interfacial processes connected with time constants ranging from minutes down to microseconds, and its applications include corrosion, characterization of thin films and coatings, batteries, semiconductor electrodes, sensors, biological systems, and many more.

It is worth emphasizing that, usually, electrochemical experiments are conducted in the presence of a large excess of supporting electrolyte to ensure charge transfer in a solution by the ions of the supporting electrolyte, but not by charged reactants and products (their migration in such conditions can be neglected). In this respect, Brudzisz et al. [140] study the effect of different supporting electrolytes (NaClO_4_, NaH_2_PO_4_, and Na_2_HPO_4_) on the electrochemical reduction of chloroform at a silver electrode in aqueous solutions. It was demonstrated that a type of anion strongly affects the potential and current density of the chloroform reduction peak. The electroreduction of chloroform at Ag occurs via a concerted mechanism, and the highest activity of Ag toward CHCl_3_ reduction was observed in NaClO_4_.

A complex electrochemical characterization of the screen-printed electrodes modified with AuNPs and the peptide PEPTIR-1.0 (sequence QKVNIDELGNAIPSGVLKDD) was performed by electrochemical techniques such as CV, SWV, and EIS [131]. Using the EIS technique, the above-mentioned electrode was tested as a potential biosensor towards detection of *Escherichia coli*. It was found that the biosensor exhibits a linear performance in the range from 0 to 500 CFU/mL, and its limits of detection and quantification are 2 and 6 CFU/mL, respectively.

Yang et al. [141] synthesized a novel and unique composite material (MnS@CoS/C@C) consisting of closed and conductive hollow polyhedral nanospheres composed of two layers of heterogeneous shells, a CoS/C inner shell and a pure C outer shell. Inside the hollow polyhedral nanospheres, several MnS nanopolyhedrons were formed from MnCO_3_ polyhedrons by a sulfurization process. The prepared composite was tested as an anode for lithium-ion batteries by CV, EIS, and galvanostatic charge–discharge measurements. It was demonstrated that the MnS@CoS/C@C composite exhibits superior Li storage performance in terms of reversible capacity, cycling performance, and rate capability.

## 3. Conclusions

Nanostructured materials have attracted much attention from scientists and engineers due to their unique and novel physical and chemical properties. The development of available and cheap methods for their synthesis and characterization techniques contributed not only to the miniaturization of new devices and systems, but also opened up new possibilities for modern technologies. Current electrochemical techniques used for the fabrication of thin films and nanostructured materials based on both cathodic and anodic processes offer very simple and effective procedures for the synthesis of a great variety of materials, but have also some limitations when manipulating both at the macro- and nanoscale simultaneously. These obstacles and hindrances may change with further development of synthesis methods and upcoming advanced nanotechnologies.
